# Myeloid-derived growth factor in diseases: structure, function and mechanisms

**DOI:** 10.1186/s10020-024-00874-z

**Published:** 2024-07-19

**Authors:** Peng Chen, Xiaohui Huang, Weiwen Li, Weixing Wen, Yue Cao, Jiahuan Li, Yuli Huang, Yunzhao Hu

**Affiliations:** 1 Department of Cardiology, Shunde Hospital, Southern Medical University, The First People’s Hospital of Shunde, NO. 1 Jiazi Road, Lunjiao, Shunde District, Foshan City, Guangdong 528308 China; 2 The George Institute for Global Health, Faculty of Medicine, University of New South Wales, Sydney, NSW2006 Australia; 3 Guangdong Provincial Key Laboratory of Cardiac Function and Microcirculation Research, Guangzhou, 510000 China; 4 Medical Research Center, Shunde Hospital, Southern Medical University, The First People’s Hospital of Shunde, NO. 1 Jiazi Road, Lunjiao, Shunde District, Foshan City, Guangdong 528308 China

**Keywords:** Myeloid-derived growth factor, Cardiovascular diseases, Metabolic disorders, Renal disease, Autoimmune/inflammatory disorders, Cancers

## Abstract

Myeloid-derived growth factor (MYDGF) is a novel secreted protein with potent antiapoptotic and tissue-repairing properties that is present in nearly 140 human tissues and cell lines, with the highest abundance in the oral epithelium and skin. Initially, MYDGF was found in bone marrow-derived monocytes and macrophages for cardioprotection and repair after myocardial infarction. Subsequent studies have shown that MYDGF plays an important role in other cardiovascular diseases (e.g., atherosclerosis and heart failure), metabolic disorders, renal disease, autoimmune/inflammatory disorders, and cancers. Although the underlying mechanisms have not been fully explored, the role of MYDGF in health and disease may involve cell apoptosis and proliferation, tissue repair and regeneration, anti-inflammation, and glycolipid metabolism regulation. In this review, we summarize the current progress in understanding the role of MYDGF in health and disease, focusing on its structure, function and mechanisms. The graphical abstract shows the current role of MYDGF in different organs and diseases (Fig. 1).

## Introduction

Myeloid-derived growth factor (MYDGF), also known as the open reading frame on chromosome 19 (C19ofr10), was originally cloned from a bone marrow-derived stromal cell line and named interleukin-25 (IL-25/SF20) because of its perceived ability to promote lymphocyte proliferation by Tulin and colleagues in 2001 (Tulin et al. 2001). However, this proliferative activity could not be reproduced, so the paper was withdrawn in 2003 (Tulin et al. 2003). Although it has also been suggested to be named IL-27 (a heterodimeric cytokine composed of EBI3 and p28 protein), this protein does not correspond to the IL-27 protein described in the literature (Pflanz et al. 2002). It is a product of chromosome 19 ORF 10, so it was named C19orf10 (Dasuri et al. 2004). C19orf10 was later found in bone marrow monocyte-macrophages expressing high levels of C-X-C motif chemokine receptor 4, and due to its potent cardiomyocyte-protective and angiogenic activity in myocardial infarction, it was formally named myeloid-derived growth factor (MYDGF) (Korf-Klingebiel et al. 2015). Subsequent proteomic studies showed that the protein was secreted in six different cell lines (Dasuri et al. 2004; Weeraphan et al. 2012; Bailey et al. 2011; Sunagozaka et al. 2011; Wilkerson et al. 2016; Wang et al. 2004).

The historical background of C19orf10 has led to considerable confusion in the literature and databases regarding the name of this protein. A literature search for either of the two names (MYDGF and C19orf10) revealed overwhelmingly different results for IL-25 (IL-17E) and IL-27. However, C19orf10 is not similar to either IL-25 or IL-27. The IL-25 annotation has been changed in the NCBI databases, and the HUGO (Human Genome Organization) Gene Nomenclature Committee (London, UK) has indicated that IL-25 and IL-27 will not be used as aliases for MYDGF (Weiler et al. 2007). Human MYDGF (hMYDGF) consists of 142 residues with a theoretical mass of 15.8 kDa and three possible splice variants (Weiler et al. 2007; Bortnov et al. 2018, 2019). The circulating concentration of hMYDGF in adults is approximately 3.3 ng/ml, and the plasma concentration of MYDGF is elevated during acute myocardial infarction and severe aortic stenosis (Korf-Klingebiel et al. 2021; Polten et al. 2019). To date, this protein has been implicated in cardiovascular disease, metabolic disorders, inflammatory disease, kidney disease and cancer and plays different roles in these diseases. In this review, we summarize the current progress in understanding the role of MYDGF in health and disease, focusing on its structure, function and mechanisms.

**Fig. 1:**
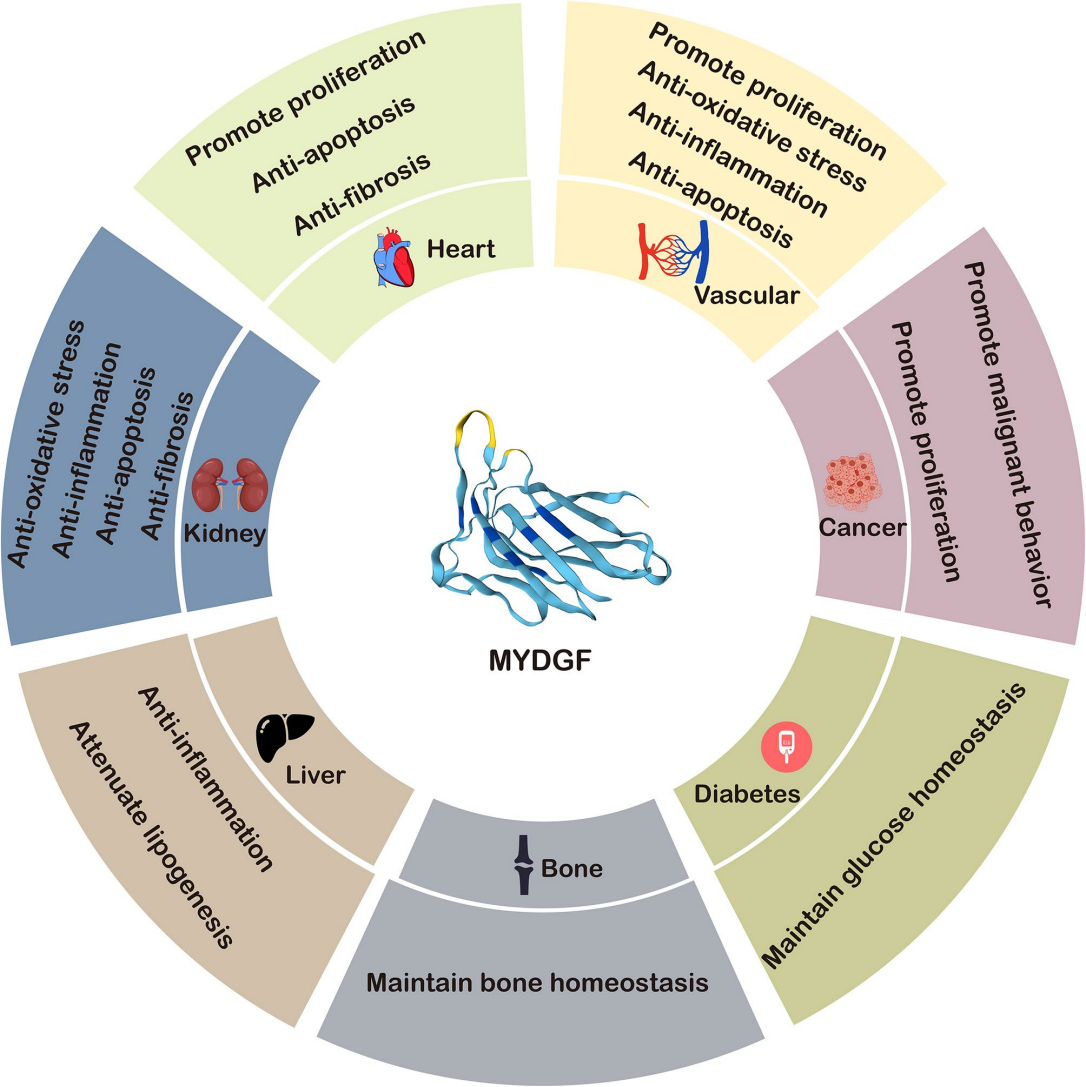
Graphical abstract showing the role of MYDGF in different organs and diseases

## The structure of MYDGF

The crystal and solution structures of MYDGF have been studied. The structure of [U-13 C, 15 N]-hMYDGF in a pH = 6 solution was determined using protein nuclear magnetic resonance (NMR) techniques by Bortnov V et al. (Bortnov et al. 2019). hMYDGF is a 142-residue protein with a global fold of 10 β-strands comprising three antiparallel β-sheets (β1–10) and an α-helical turn (α1). The largest β-sheet (β1, β4, β5, β10, and β7) is linked to a smaller β-sheet (β2, β3, and β6) by a disulfide bridge to form a β-sandwich (Bortnov et al. 2019).

**Fig. 2:**
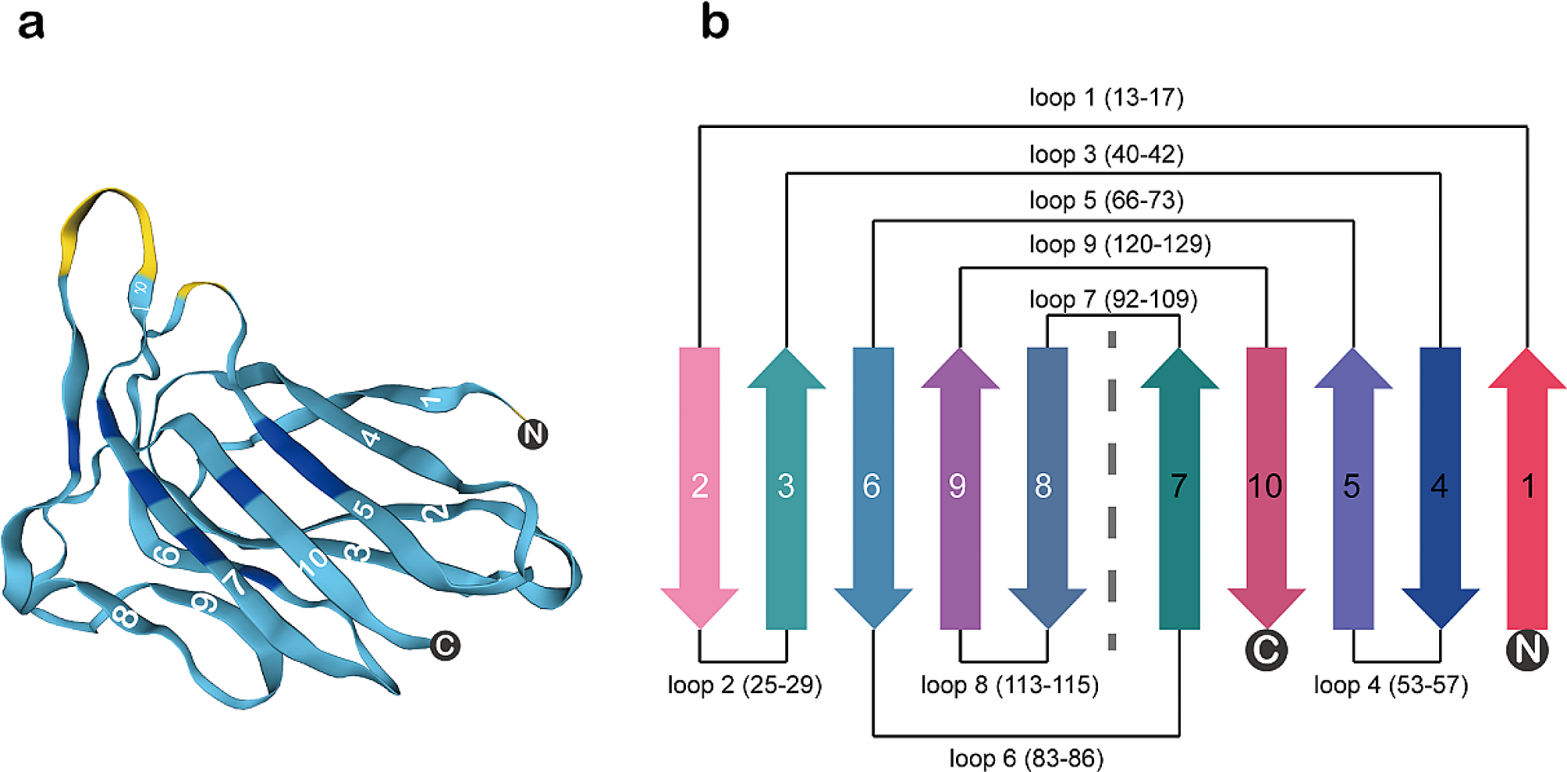
MYDGF structure and topology diagram. **a** The structure is made up of ten β-strands (β1–β10) that comprise three antiparallel β-sheets (β1–10) and an α-helical turn (α1). **b** Topology diagram of β-strand connectivity. Each arrow represents a β-sheet (β1–10). β1 and β10 connect the N and C peptide termini, respectively. β1 and β2 form loop 1 (5 amino acids in length), β2 and β3 form loop 2 (5 amino acids in length), β3 and β4 form loop 3 (3 amino acids in length), β4 and β5 form loop 4 (5 amino acids in length), β5 and β6 form loop 5 (8 amino acids in length), β6 and β7 form loop 6 (6 amino acids in length), β7 and β8 form loop 7 (18 amino acids in length), β8 and β9 form loop 8 (3 amino acids in length), and β9 and β10 form loop 9 (10 amino acids in length)

Similar to the solution structure, the crystal structure of MYDGF consists of 10 antiparallel β-strands that form two face-to-face aligned β-sheets to form a β-sandwich (Ebenhoch et al. 2019). The loop structure of MYDGF is highly asymmetric. One end of the β-sheets with short hairpin-like loops is called the bottom face, and the other end with three elongated loops (loops 5, 7, and 9) is the top face (Ebenhoch et al. 2019). The β-sheet plane consisting of β-strands 1, 4, 5, 10, and 7 is named the front face, while the β-sheet plane consisting of β-strands 2, 3, 6, 9, and 8 is the back face (Ebenhoch et al. 2019). The N- and C-termini are located at the bottom face, the N-terminus contains a 31-residue-long signal peptide, and the C-terminus contains a KDEL-like ER (endoplasmic reticulum) retention sequence (Bortnov et al. 2018). The N-terminal signaling peptide can target MYDGF to enter the classical secretory pathway through ER membrane translocation (Almagro Armenteros et al. 2019) and exerts its potent protective effects. Based on the structure of MYDGF, it is predicted that the receptor epitope of MYDGF may be at the front or protruding part of the upper surface of the MYDGF structure. Try73 may be a key residue involved in the interaction of MYDGF with the receptor (Ebenhoch et al. 2019). The structure and topology diagram of MYDGF are shown in Fig. 2.

Bone marrow-derived monocytes and macrophages are the major secretory cells of MYDGF. Bortnov V et al. explored whether eosinophils are another major secretory organ of MYDGF. They unexpectedly found that HEK293 cells transfected with MYDGF lacking the last two residues secreted large amounts of the protein into the extracellular space. However, HEK293 cells transfected with full-length recombinant human MYDGF retained the vast majority of MYDGF intracellularly (Bortnov et al. 2018). Furthermore, intracellular MYDGF in eosinophils was found to colocalize extensively with the ER marker P4HB in central patches around the nucleus and between the nuclear lobes (Bortnov et al. 2018). This finding demonstrated that MYDFG is an ER-resident protein and that the C-terminal RTEL sequence of human MYDGF is required for MYDGF retention in the ER.

Although a search of different databases revealed that homologs of MYDGF exist throughout the biological kingdom, it does not belong to any known cytokine or growth factor family based on its primary amino acid sequence (Ebenhoch et al. 2019), suggesting that it may have other specialized biological functions.

## MYDGF in cardiovascular diseases

### MYDGF in myocardial infarction (MI)

In neonatal rat ventricular myocytes (NRVMs) after ischemia and reperfusion (IR) injury, MYDGF enhances the phosphorylation of AKT at T308 and S473, promotes the phosphorylation of BAD (S136) and BAX (S184), and decreases the levels of cytosolic cytochrome C, cleaved caspase9 and the enzymatic activities of the effectors caspase3 and caspase7 (Korf-Klingebiel et al. 2015). The cytoprotective effect of MYDGF on NRVM was eliminated by the PI3K inhibitor LY294002, suggesting that MYDGF mediates the cytoprotective effect of NRVM after IR injury through the PI3K-AKT signaling pathway (Korf-Klingebiel et al. 2015).

MYDGF can promote the proliferation of endothelial cells by increasing the rapid phosphorylation of MAPK1 and MAPK3 and the phosphorylation of STAT3 at S727, significantly upregulating the expression of cyclin D1 (Korf-Klingebiel et al. 2015; Du et al. 2022; Zhao et al. 2020). MYDGF was found to promote cardiomyocyte proliferation by activating the c-Myc/FoxM1 pathway and promoting cardiac regeneration after heart injury in neonatal and adult mice (Wang et al. 2020a, b). In addition, the main site of MYDGF expression in the neonatal mouse myocardium is not mononuclear macrophages but endothelial cells (Wang et al. 2020a, b).

It was recently reported that MYDGF can attenuate IR-induced apoptosis of cardiac microvascular endothelial cells by regulating ER oxidative stress after IR injury (Wang et al. 2022).

When MI occurs, MYDGF-deficient mice exhibit a larger infarct size, more cardiomyocyte apoptosis, reduced endothelial cell proliferation, and diminished angiogenic responses, which are attenuated by the administration of the MYDGF protein (Korf-Klingebiel et al. 2015). Delaying the treatment until 6 h after reperfusion still reduced scar size and improved systolic function (Korf-Klingebiel et al. 2015), suggesting that in addition to the early effects on infarct size, the late effects of MYDGF on infarct healing are also important for functional recovery. Intramyocardial injection of an MYDGF-loaded citrate-containing hydrogel in MI mice significantly reduced scar formation and infarct size, increased wall thickness and neovascularization, and improved cardiac function (Yuan et al. 2019).

### MYDGF in atherosclerosis

Atherosclerosis is a chronic inflammatory disease of the arterial wall that can cause ischemic heart disease, stroke, and peripheral vascular disease. Endothelial dysfunction is an early pathophysiological change in the development of atherosclerosis, and MYDGF has been shown to promote endothelial cell proliferation (Korf-Klingebiel et al. 2015). MYDGF can attenuate palmitic acid-induced endothelial inflammation, apoptosis, permeability, and adhesion responses (Meng et al. 2021). Bone marrow MYDGF-specific knockout mice exhibit severe endothelial damage, significantly increased inflammation, and increased susceptibility to atherosclerosis (Meng et al. 2021). Bone marrow transplantation or bone marrow-specific overexpression of MYDGF attenuated leukocyte homing in the aortas of atherosclerotic mice and ameliorated atherosclerosis by reducing inflammation and endothelial cell damage via the PKCδ/MAP4K4/NF-κB pathway (Meng et al. 2021). Endothelial dysfunction leads to the accumulation of circulating low-density lipoprotein cholesterol (LDL-C) in the subendothelial space, which is one of the early stages of atherosclerosis. Inflammatory cell-derived MYDGF deficiency aggravated endothelial LDL-C transcytosis, drove LDL-C uptake by the artery wall, and thus exacerbated atherosclerosis, whereas MYDGF inhibited MAP4K4 phosphorylation, enhanced AKT-1 activation, reduced the FoxO3a signaling cascade, and inhibited LDL transcytosis to protect against atherogenesis (Xu et al. 2023). In addition, MYDGF relieves neointimal formation in carotid arteries in response to balloon injury in rats and suppresses vascular smooth muscle cell dedifferentiation induced by PDG-BB via the sphingosine-1-phosphate receptor 2-RhoA-actin monomers (G-actin)/actin filaments (F-actin)-MRTF-A signaling pathway (Yang et al. 2024a, b). This finding suggested that MYDGF may be used for the treatment of neointimal formation and restenosis.

### MYDGF in heart failure

Heart failure is the end manifestation of various heart diseases. MYDGF treatment alleviates systolic dysfunction in mice with MI (Korf-Klingebiel et al. 2015; Yuan et al. 2019). Moreover, MYDGF knockout mice exhibit more severe left ventricular hypertrophy and contractile dysfunction during pressure overload (Korf-Klingebiel et al. 2021). In contrast, MYDGF conditional transgenic overexpression or MYDGF protein injection can inhibit G protein-coupled agonist-induced hypertrophy and augment Sarco/endoplasmic reticulum Ca(2+)-ATPase (SERCA2a) expression in cardiomyocytes by enhancing Pim-1 proto-oncogene serine/threonine kinase (PIM1) expression and activity, thereby attenuating pressure overload-induced ventricular hypertrophy and dysfunction (Korf-Klingebiel et al. 2021). Interestingly, enhanced myocardial MYDGF expression after transverse aortic constriction (TAC) was accompanied by a sustained increase in plasma MYDGF (Korf-Klingebiel et al. 2021), suggesting that MYDGF is released into the extracellular space via paracrine secretion. However, the underlying mechanisms leading to MYDGF release are not clear. Trychta et al. reported that calcium depletion in the ER leads to the secretion of ER-resident proteins, including MYDGF (Trychta et al. 2018). Therefore, we speculate that the increase in MYDGF in the circulation after TAC may be due to decreased SERCA2a expression in cardiomyocytes in response to pressure overload and that calcium depletion triggers the secretion of resident ER luminal proteins.

Cardiac fibrosis is a pathology that occurs after injury and during aging. The persistence of fibrosis reduces tissue compliance and accelerates heart failure progression. Recent studies have reported that MYDGF is coexpressed with genes regulated in TGFβ1-induced fibrosis models and has been identified as a potential therapeutic target for cardiac fibrosis (Hinderer and Schenke-Layland 2019; Wilson et al. 2022). MYDGF may exert antifibrotic effects through the TGFβ1 pathway and inhibit the progression of heart failure.

Therefore, MYDGF may play a beneficial role in cardiovascular disease through a variety of mechanisms, including improving endothelial cell function, reducing cardiomyocyte apoptosis, promoting cardiomyocyte regeneration, and inhibiting cardiac fibrosis. The role of MYDGF in heart disease described above is summarized in Table 1.

**Table 1:** MYDGF in cardiovascular diseases

Disease	Model	Effect	Pathway	Reference
Normal	HUVECs	Promote proliferation	MAPK1/3-STAT3/cyclinD1	(Korf-Klingebiel et al. 2015; Du et al. 2022; Zhao et al. 2020)
IR injury	NRVM and HUVECs	Inhibit apoptosis	PI3K/AKT	(Korf-Klingebiel et al. 2015; Du et al. 2022)
Hypoxia/reoxygenation	CMECs	Regulate oxidative stress in ERS, reduce apoptosis	Remain unknown	(Wang et al. 2022)
MI	MYDGF-/- mice	Reduce scar size, improve systolic function and heart regeneration	Remain unknown	(Korf-Klingebiel et al. 2015; Wang et al. 2020a, b; Yuan et al. 2019)
Apical resection	MYDGF-/- mice	Improve cardiomyocyte proliferation and neonatal heart regeneration	c-Myc/FoxM1	(Wang et al. 2020a, b)
Atherosclerosis	MYDGF-/- mice	Attenuate inflammation and endothelial cell damage, inhibits LDL-C transcytosis across endothelium	PKCδ/MAP4K4/NF-κB, MAP4K4/Akt1/FoxO3a	(Meng et al. 2021; Xu et al. 2023)
Heart failure	MYDGF-/- mice	Attenuate pressure overload-induced hypertrophy and dysfunction	G protein-coupled receptor agonist and PIM1/SERCA2a	(Korf-Klingebiel et al. 2021)
Artery balloon injury	VSMC and Sprague-Dawley rats	Relieve neointimal formation and suppress VSMC dedifferentiation	S1PR2-RhoA-G/F-actin-MRTF-A	(Yang et al. 2024a, b)

## MYDGF in metabolic disorders

Since the formal name MYDGF was used, the effect of MYDGF on cardiovascular disease has been explored. Recent studies have also shown that MYDGF is involved in multiple metabolic disorders, including glucose and lipid metabolism, non-alcoholic fatty liver disease (NAFLD), and bone metabolism (Table 2).

**Table 2:** MYDGF in diseases associated with metabolic disorders

Disease	Model	Effect	Pathway	Reference
Diabetes	MYDGF-/- mice	Maintain glucose homeostasis by inducing GLP-1 production and secretion and improve glucose tolerance and lipid metabolism	PKA/GSK-3β/β-catenin, MAPK/MEK/ERK	(Wang et al. 2020a, b)
Non-alcoholic fatty liver disease	Myeloid cell-specific MYDGF knockout mice	Alleviate hepatic inflammation, lipogenesis, and steatosis	PKC/IKKβ/NF-κB	(Ding et al. 2023)
Osteoporosis	Myeloide cell-specific MYDGF knockout mice	Inhibit osteoclastogenesis and promote osteoblast differentiation	PKCβ/NF-κB, MAPK1/3-STAT3	(Xu et al. 2022)

MYDGF is secreted in mouse fibroblasts during adipocyte differentiation, suggesting that it may be involved in adipogenesis and adipose tissue development (Wang et al. 2004). In type 2 diabetes, MYDGF promotes the production and secretion of GLP-1 by intestinal L-cells through the PKA/GSK-3β/β-catenin and MAPK/MEK/ERK signaling pathways and improves insulin resistance, glucose tolerance, and lipid metabolism (Wang et al. 2020a, b). NAFLD represents a spectrum of diseases, including simple steatosis, steatohepatitis, and cirrhosis (Piccinin et al. 2019). NAFLD is associated with inflammation, insulin resistance (IR) and dyslipidemia, and metabolic disorders have been shown to be strongly associated with NAFLD (Wang et al. 2018; Yu et al. 2018). Myeloid-specific knockdown of MYDGF exacerbates inflammation, adipogenesis, and steatosis in NAFLD mice (Ding et al. 2023). Bone marrow transplantation or myeloid-specific overexpression of MYDGF can ameliorate NAFLD through the PKC/IKKβ/NF-κB signaling pathway (Ding et al. 2023). In addition, MYDGF can significantly attenuate palmitic acid-induced inflammation in Kupffer cells and promote the polarization of macrophages toward the M2 phenotype (Ding et al. 2023). Therefore, MYDGF may ameliorate NAFLD by inhibiting hepatocyte inflammation and modulating lipid metabolism.

Accumulating evidence suggests that some growth factors, such as GDF11 (Liu et al. 2016), platelet-derived growth factor-BB (PDGF-BB) (Xie et al. 2014) and vascular endothelial growth factor (VEGF), play a central role in maintaining the balance of the remodeling cycle that links bone resorption to bone formation (Hu and Olsen 2016). Recent studies have reported that MYDGF deficiency leads to bone loss and decreased bone strength in young and old mice (Xu et al. 2022). MYDGF inhibits osteoclast differentiation through the PKCβ/NF-κB signaling pathway and promotes osteoblast differentiation through the MAPK1/3-STAT3 signaling pathway, which in turn facilitates the healing of bone defects and prevents ovariectomy-induced bone loss and osteoporosis (Xu et al. 2022).

## MYDGF in kidney diseases

MYDGF can ameliorate renal dysfunction induced by I/R injury, attenuate oxidative stress, inhibit inflammation and reduce renal tubular cell apoptosis through activation of the AKT pathway (Wang et al. 2023a, b). Podocytes are highly specialized and terminally differentiated epithelial cells that are key components of the glomerular filtration barrier (Torban et al. 2019). Podocyte-specific deletion of MYDGF exacerbates podocyte injury and proteinuria in mice with ADR-induced focal segmental glomerulosclerosis (Zhan et al. 2022). Under normal physiological conditions, podocytes are in a post-mitotic state, and podocytes re-enter the mitotic phase after stress, which is known as mitotic catastrophe, leading to podocyte death and glomerular damage (Nagata 2016). In adriamycin-induced podocyte injury, MYDGF can inhibit podocyte reentry into the cell cycle through the RUNX2/p27/cyclinA/CDK2 signaling pathway and attenuate podocyte injury (Zhan et al. 2022). In addition, MYDGF can alleviate renal capillary thinning, hypoxia, renal fibrosis and tubular atrophy in UUO (unilateral ureteral obstruction) and adenine-induced CKD rats (Du et al. 2022). Diabetic kidney disease (DKD) is one of the most common complications of diabetes mellitus. Moreover, MYDGF deficiency led to more severe podocyte and glomerular injury and increased proteinuria in diabetic kidney disease (DKD) mice (He et al. 2020). The administration of MYDGF can ameliorate glomerular injury and proteinuria, regulate glucose metabolism, and inhibit podocyte apoptosis through the AKT/BAD signaling pathway (Zhan et al. 2022; He et al. 2020). There is evidence that GLP-1 can ameliorate DKD (Mann et al. 2017; Fujita et al. 2014), and MYDGF can stimulate the production and secretion of GLP-1 by intestinal L cells in diabetic mice (Wang et al. 2020a, b), which may be one of the important mechanisms for its protective effect against DKD. The roles and mechanisms of MYDGF in kidney diseases are summarized in Table 3.

**Table 3:** MYDGF in kidney diseases

Disease	Model	Effect	Pathway	Reference
Renal IR injury	C57BL/6 mice, HK-2 and Human Kidney Cell	Improve renal function, reduce oxidative stress, inflammation, and apoptosis	The phosphorylation Akt	(Wang et al. 2023a, b)
Focal segmental glomerulosclerosis	Podocyte-specific MYDGF knockout mice	Protected podocytes against mitotic catastrophe	RUNX2/P27/cyclinA/CDK2	(Zhan et al. 2022)
Diabetic kidney disease	Podocyte-specific MYDGF knockout mice, MYDGF-/- mice	Protected podocytes against mitotic catastrophe, preserve nephrin expression and inhibit podocyte apoptosis	RUNX2/P27/cyclinA/CDK2, Akt/BAD	(Zhan et al. 2022; He et al. 2020)
CKD	C57BL/6J mice, Wistar rats	Alleviate capillary rarefaction, hypoxia, renal fibrosis, and tubular atrophy	Remain unknown	(Du et al. 2022)

## MYDGF in the inflammatory response

MYDGF may also be involved in a number of autoimmune diseases. MYDGF was identified in synovial fibroblast-like synovial cell proteins from patients with rheumatoid arthritis, which were distributed in the perivascular and synovial lining in synovial sections from rheumatoid arthritis and osteoarthritis patients (Dasuri et al. 2004; Weiler et al. 2007). In zebrafish, tissue damage induces increased expression of MYDGF, which inhibits neutrophil accumulation through the HIF-1α pathway and limits neutrophil inflammation in response to tissue injury (Houseright et al. 2021). In addition, MYDGF can inhibit the expression of pro-inflammatory factors and chemokines in gingival fibroblasts induced by high glucose, which provides a new treatment for periodontal disease (Gao et al. 2023). Previous studies have demonstrated the role of MYDGF in promoting M2 macrophage polarization (Ding et al. 2023). In this regard, MYDGF was able to regulate macrophage polarization and inhibit the NF-κB and MAPK pathways to reduce inflammation and ameliorate colitis (Yang et al. 2024a, b).

Recent studies have reported that the level of MYDGF is increased in NK cells from HIV-1-infected people whose viral load is undetectable in the absence of antiretroviral therapy (Wang et al. 2023a, b). Like amphiregulin (AREG), which is expressed by NK cells, the MYDGF locus also contains a binding site for transcription factor 7 (Wang et al. 2023a, b). Further investigation into the effect of AREG and MYDGF on NK cells may help to elucidate the mechanisms underlying chronic inflammation and increased risk of cardiovascular disease in HIV-1 infected people (Wang et al. 2023a, b). In addition, 16 differentially expressed genes, including MYDGF, were identified by transcriptome analysis of healthy adults who exhibited a complete seroprotective effect after receiving a single dose of influenza vaccine (Tawfik et al. 2024). This reveals that MYDGF plays an important role in the construction of stable and long-term immune responses in the body after vaccination.

## MYDGF in cancer

MYDGF has potent anti-apoptotic and tissue repair effects on many diseases, however, these beneficial effects in cancer promote tumor cell development and further deterioration (Table 4). The secretion of the MYDGF protein was found to be increased in cholangiocarcinoma cells in a hollow fiber bioreactor culture system (Weeraphan et al. 2012). Serial analysis of gene expression showed that MYDGF was highly expressed in hepatocellular carcinoma (HCC), significantly and positively correlated with AFP, and promoted the proliferation of HCC cell lines through the AKT/MAPK pathway (Sunagozaka et al. 2011). MYDGF can promote self-renewal of liver tumor stem cells and tumor angiogenesis, induce macrophage chemotaxis into tumor tissues and release a variety of inflammatory factors, which ultimately aggravate inflammation in the tumor microenvironment and accelerate HCC progression (Wang et al. 2021). After partial hepatectomy, the hepatic vasculature experiences shear stress due to increased blood flow to the remaining liver (Michalopoulos 2010). This shear stress induces vasodilation and concomitant mechanical stretching of hepatic vascular endothelial cells. This leads to increased secretion of MYDGF protein by hepatic endothelial cells, which promotes hepatocyte proliferation through activation of MAPK and STAT3 (Große-Segerath et al. 2024).

**Table 4:** Role of MYDGF in cancer

Disease	Model	Effect	Pathway	Reference
Hepatocelluar carcinoma	Human liver cancer cell lines	Enhance the proliferation of liver cancer cells Aggravate inflammation of tumor microenvironment, accelerate HCC progression	Akt/MAPK Remain unknown	(Sunagozaka et al. 2011) (Wang et al. 2021)
Kidney Renal Clear cell Carcinoma	Human renal cell carcinoma cell lines	Promote cell viability, proliferation, migration and invasion	ZO-1 and PTEN/Akt	(Lu et al. 2021)
Bladder cancer	Human bladder cancer cell lines	Promote malignant behaviors and EMT of human bladder carcinoma cells	PI3K/Akt and Wnt/β-catenin	(Li et al. 2021)

Deep transcriptional sequencing data from KIRC (kidney renal clear cell carcinoma) samples showed that the level of MYDGF was greater in KIRC tissue than in para-carcinoma tissues (Zhou et al. 2010). MYDGF can enhance KIRC cell viability and proliferation, promote cell migration and invasion, and facilitate KIRC progression through the ZO-1 and PTEN/Akt signaling pathways (Lu et al. 2021). Similarly, transcriptome analysis of bladder cancer tissues revealed high expression of MYDGF (Zhu et al. 2011), which can promote cancer cell proliferation, migration and invasion, and promote malignant behavior and epithelial-mesenchymal transition (EMT) in human bladder cancer by regulating the PI3K/AKT and Wnt/β-catenin pathways (Li et al. 2021).

Biodegradable guanidinium-functionalized polycarbonates are potential cancer chemotherapeutic agents (Park et al. 2018). It can interact specifically with cancer cells, leading to their death through apoptosis (Zhong et al. 2019). However, the exact molecular targets of such anticancer polycarbonates are unknown. Recent studies have identified MYDGF as a major protein target of polycarbonates, which could be a potential new target for macromolecular chemotherapy development (Sim et al. 2024).

## Conclusions

MYDGF is rapidly gaining attention as a significant regulator of various diseases, and is considered as a novel secreted protein with strong anti-apoptosis and tissue repair abilities. In addition, MYDGF can improve disease through regulating proliferation, tissue repair and regeneration, inflammation and glycolipid metabolism. Moreover, to better explore the unknown function of MYDGF in disease, methods for detecting MYDGF and engineering techniques for extending the half-life of MYDGF have been developed. However, while current discoveries seem promising, several vital problems remain to be clarified, such as the lack of large-sample clinical studies demonstrating that MYDGF presumably is a biomarker for disease diagnosis and prognosis, the receptor of MYDGF remains unknown, and many undiscovered functions and mechanisms of MYDGF remain to be explored. In summary, we believe that MYDGF might become a promising therapeutic target for treating diseases.

**Fig. 3:**
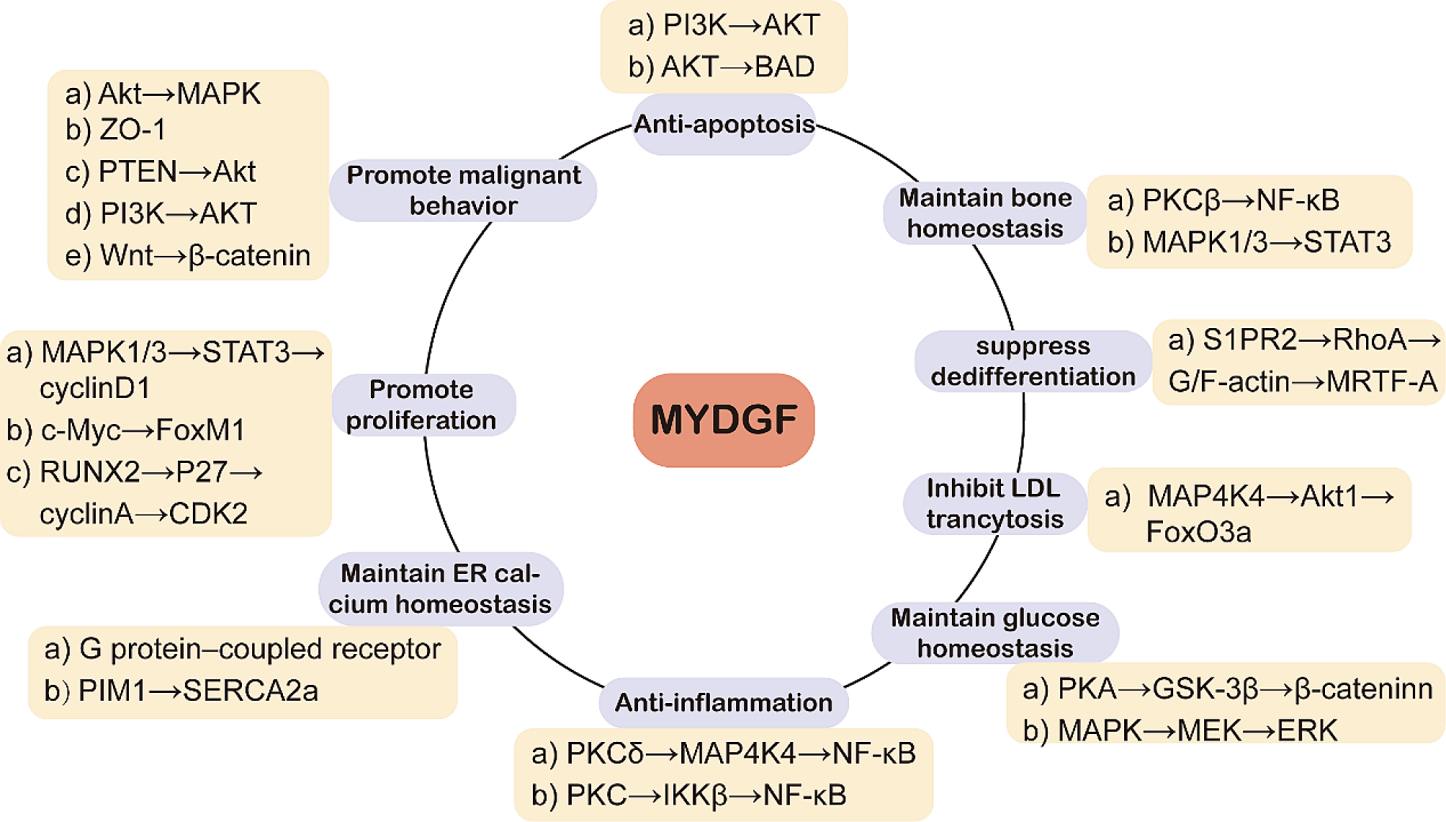
Mechanisms and signaling pathways affected by MYDGF in disease

## Data Availability

Not applicable.

## Abbreviations

β-catenin: Catenin beta 1
AKT: Akt kinase
BAD: BCL2 associated agonist of cell death
c-Myc: Transcriptional regulator Myc-like
CDK2: Cyclin dependent kinase 2
CKD: Chronic kidney disease
CMECs: Cardiac microvascular endothelial cells
EMT: Epithelial-mesenchymal transition
ER: Endoplasmic reticulum
ERK: Extracellular regulated protein kinases
ERS: Endoplasmic reticulum stress
FoxM1: Forkhead box M1
FoxO3a: Forkhead box O3A
GLP-1: Glucagon like peptide 1
GSK-3β: Glycogen synthase kinase 3 beta
HCC: Hepatocellular carcinoma
HK-2: Human proximal tubular epithelial cells
HUVECs: Human Umbilical Vein Endothelial Cells
IKKβ: I-kappaB kinase beta
IR: Ischemia and reperfusion
LDL-C: Low-density lipoprotein cholesterol
MAP4K4: Mitogen-activated protein kinase kinase kinase kinase 4
MAPK1: Mitogen-activated protein kinase 1
MAPK3: Mitogen-activated protein kinase 3
MAPK: Mitogen activated kinase-like protein
MEK: MAP kinse-ERK kinase
MI: Myocardial infarction
MRTF-A: Myocardin related transcription factor A
NF-κB: Nuclear factor kappa B
NRVMS: Neonatal rat ventricular myocytes
p27: P27 protein
PI3K: Phosphatidylinositol 3-kinase
PIM1: Pim-1 proto-oncogene, serine/threonine kinase
PKA: CAMP dependent protein kinase
PKCβ: Protein kinase C beta
PKCδ: Protein kinase C delta
PTEN: Phosphatase and tensin homolog
RhoA: Ras homolog family member A
RUNX2: RUNX family transcription factor 2
SERCA2a: Sarco/endoplasmic reticulum Ca(2+)-ATPase
SIPR2: Sphingosine-1-phosphate receptor 2
STAT3: Signal transducer and activator of transcription 3
VSMC: Vascular smooth muscle cell
Wnt: Protein Wnt-2
ZO-1: Tight junction protein 1
